# Peroral cholangioscopy‐guided forceps biopsy *versus* fluoroscopy‐guided forceps biopsy for extrahepatic biliary lesions

**DOI:** 10.1002/jgh3.12403

**Published:** 2020-08-07

**Authors:** Takumi Onoyama, Wataru Hamamoto, Yuri Sakamoto, Shiho Kawahara, Taro Yamashita, Hiroki Koda, Soichiro Kawata, Yohei Takeda, Kazuya Matsumoto, Hajime Isomoto

**Affiliations:** ^1^ Division of Gastroenterology and Nephrology, Department of Multidisciplinary Internal Medicine Tottori University Faculty of Medicine Yonago Japan

**Keywords:** accuracy, biopsy, cholangiocarcinoma, Peroral cholangioscopy, propensity score matching

## Abstract

**Background and Aim:**

Endoscopic retrograde cholangiopancreatography (ERCP)‐related tissue acquisition, including fluoroscopy‐guided forceps biopsy (F‐FB), is a common technique in diagnosing indeterminate biliary lesions. Recently, peroral cholangioscopy (POCS) and POCS‐guided forceps biopsy (POCS‐FB) has also been used for the diagnosis of indeterminate biliary lesions. However, it is uncertain which of those techniques were superior for the diagnosis of extrahepatic cholangiocarcinoma (ECC). We aimed to evaluate the diagnostic yield and safety of F‐FB for indeterminate biliary lesions compared with POCS‐FB.

**Methods:**

Patients who underwent F‐FB or POCS‐FB to evaluate indeterminate biliary lesions between October 2011 and August 2019 were enrolled retrospectively. We carried out propensity score matching to balance these clinical differences between the F‐FB group and POCS‐FB group. In the propensity score‐matched cohort, we compared the diagnostic performance of F‐FB with that of POCS‐FB based on the pathological evaluation. We also evaluate adverse events associated with F‐FB and POCS‐FB.

**Results:**

We enrolled 113 patients with biliary diseases, and 62 patients were analyzed in the propensity score‐matched cohort. Sensitivity, specificity, and accuracy of F‐FB were 82.4, 100, and 90.3%, and for POCS‐FB, those values were 83.3, 100, and 90.3%, respectively. There were no significant differences in the diagnostic performance between F‐FB and POCS‐FB. There were also no significant differences in the occurrence of adverse events between F‐FB and POCS‐FB (41.9 *vs* 29.0%, *P* = 0.289).

**Conclusions:**

The diagnostic yield of F‐FB for ECC is similar to that of POCS‐FB. POCS‐FB is not necessary for the initial pathological diagnosis of indeterminate biliary lesions.

## Graphical abstract

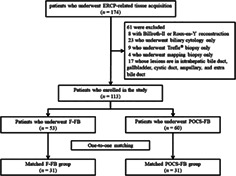

We performed a retrospective study to compare the diagnostic utility of fluoroscopy‐guided forceps biopsy for indeterminate biliary strictures and that of peroral cholangioscopy‐guided forceps biopsy in the propensity score‐matched cohort, and 62 patients were analyzed in the study. Sensitivity, specificity, and accuracy of fluoroscopy‐guided forceps biopsy (F‐FB) were 82.4, 100, and 90.3%, and for peroral cholangioscopy‐guided forceps biopsy (POCS‐FB), those values were 83.3, 100, and 90.3%, respectively. There were no significant differences in the diagnostic performance between F‐FB and POCS‐FB.

## Introduction

Extrahepatic cholangiocarcinoma (ECC), which causes biliary stricture, is one of the poor prognostic malignant diseases with a 5‐year survival rate of 20.5% (median survival time, 11.3 months).^1^ Although the early diagnosis of ECC might improve its prognosis,^2^ it is often difficult to diagnose ECC accurately because indeterminate biliary lesions include many different diseases, such as primary sclerosing cholangitis, immunoglobulin G subclass 4 (IgG4)‐associated sclerosing cholangitis, and Mirizzi syndrome.^3^

Endoscopic retrograde cholangiopancreatography (ERCP)‐related tissue acquisition, including bile aspiration cytology, biliary brush cytology, and forceps biopsy, is a standard technique to obtain specimens from indeterminate biliary lesions for pathological examinations. The specificity of the pathological evaluation of tissues obtained from indeterminate biliary lesions by ERCP‐related technique is nearly 100%. However, the sensitivity of this technique is not sufficient, with a range of 41.6–48.1%.^4^, ^5^

Recently, peroral cholangioscopy (POCS) has been widely used to diagnose indeterminate biliary lesions. POCS‐guided forceps biopsy (POCS‐FB) was also used for tissue acquisition in indeterminate biliary lesions. However, the sensitivity of POCS‐FB was also insufficient (60.1%).^6^ Moreover, a few studies reported that the sensitivity of POCS‐FB for ECC was similar to that of fluoroscopy‐guided forceps biopsy (F‐FB).^7^, ^8^ Meanwhile, there have been no randomized control trials comparing the diagnostic yield of POCS‐FB and that of F‐FB for ECC, so it is unknown which one is superior to another. In this study, we aimed to evaluate the diagnostic performance of POCS‐FB and F‐FB for indeterminate biliary lesions with propensity score‐matched analysis.

## Methods

### *Study population*

In this study, patients who underwent ERCP‐related tissue acquisition with POCS‐FB and/or F‐FB to diagnose extrahepatic biliary lesions between December 2011 and August 2019 at our hospital were enrolled retrospectively. Exclusion criteria were as follows: (i) patients who did not provide consent; (ii) patients aged 20 years or younger when endoscopic procedures were performed; (iii) patients who underwent ERCP tissue acquisition with bile cytology only and/or endoscopic scraper (Trefle); (iv) patients who had surgically altered anatomy except Billroth‐I; and (v) patients with intrahepatic biliary lesions, gallbladder lesions, ampullary lesions, and extra biliary lesions, such as pancreatic cancer. The F‐FB group was defined as including patients who underwent F‐FB as the first technique for tissue acquisition for indeterminate biliary lesion, and the POCS‐FB group was also defined in the same way. We evaluated the diagnostic performance of F‐FB and that of POCS‐FB for ECC based on the pathological evaluation. Furthermore, we compared adverse events in F‐FB with those in POCS‐FB. This study was performed according to the guidelines described in the Helsinki Declaration for biomedical research involving human participants. The study was approved by the institutional review board of Tottori University (approval number: 20A025). Informed consent was obtained from all participants using an opt‐out approach in the retrospective study.

### *Endoscopic procedure and adverse event*

A side‐viewing duodenoscope (JF260V/TJF240V/TJF290V; Olympus Optical Co., Ltd., Tokyo, Japan) was used for ERCP. We also used a 0.035‐inch hydrophilic guidewire (M00556051; Boston Scientific Corporation, Natick, MA, USA) and/or a 0.025‐inch hydrophilic guidewire (G‐260‐2545A; Olympus Optical Co., Ltd. MTA0025N48S; Medico's Hirata, Inc., Osaka, Japan. M00556700; Boston Scientific Corporation) during ERCP. If there was difficulty in inserting the cannula into the biliary tract, patients underwent a precut papillotomy with a needle knife (9 913 023 121; MTW Endoskopie W. Haag KG, Wesel, Germany).

**Figure 1:**
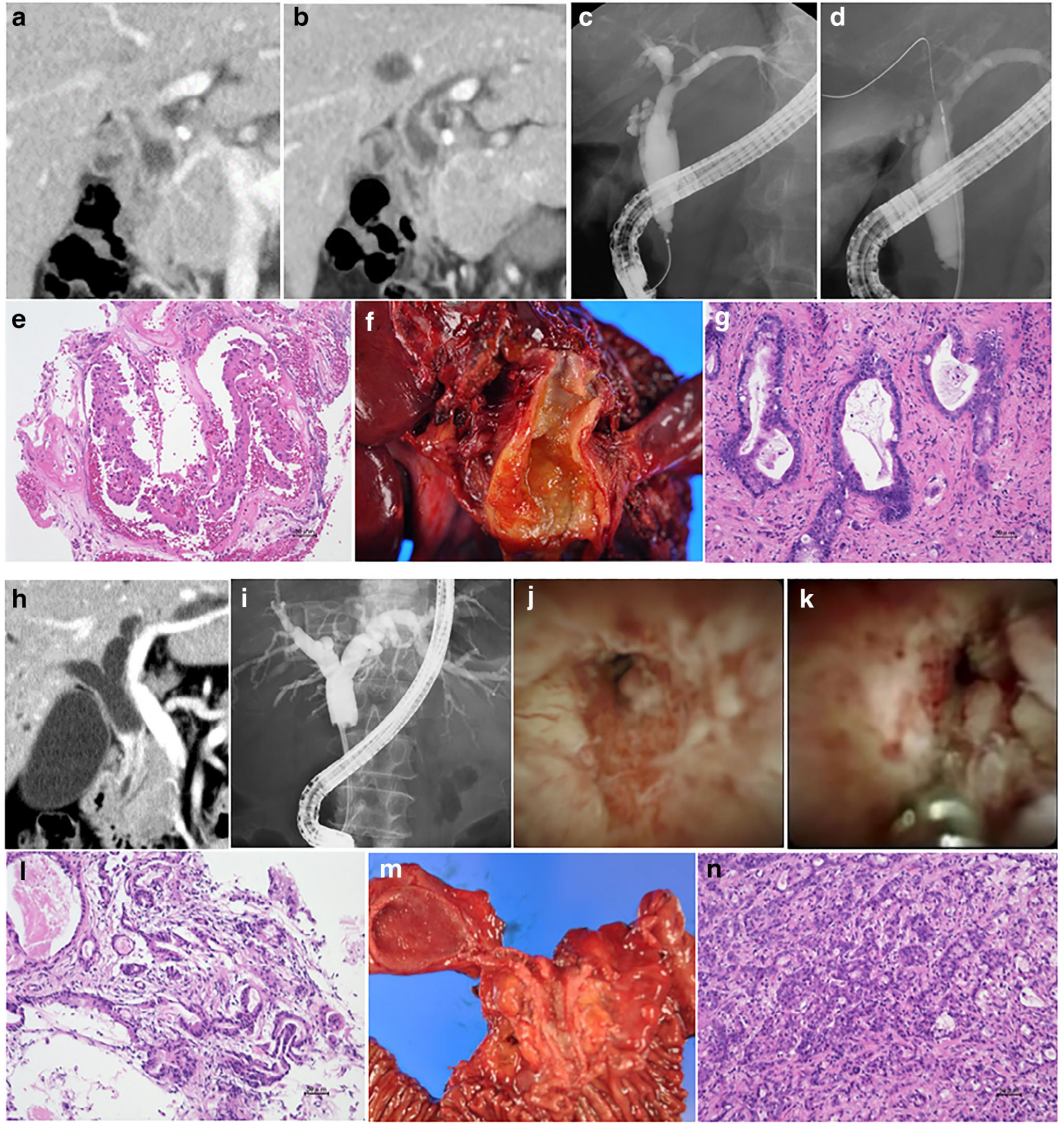
A case diagnosed as perihilar cholangiocarcinoma with fluoroscopy‐guided forceps biopsy (a–f). (a, b) Computed tomography scan showed irregular stenosis and wall thickness in the perihilar bile duct; (c) endoscopic retrograde cholangiography revealed irregular stenosis in the perihilar bile duct; (d) tissue acquisition procedure by fluoroscopy‐guided forceps biopsy was performed for the stenosis in the perihilar bile duct; (e) microscopic appearance of hematoxylin and eosin‐stained tissue sample was observed. The pathological diagnosis was adenocarcinoma. (f) This patient underwent left hepatectomy with extrahepatic bile duct resection, and (g) this patient was diagnosed as perihilar cholangiocarcinoma. Another case diagnosed as distal cholangiocarcinoma with peroral cholangioscopy (POCS)‐guided forceps biopsy (h–n). (h) Computed tomography scan showed an irregular wall thickness in the distal bile duct; (i) endoscopic retrograde cholangiography revealed stenosis in the distal bile duct; (j, k) POCS showed the irregular papillary mucosa in the distal bile duct. POCS‐guided forceps biopsy was performed for the biliary stricture in the distal bile duct; (l) hematoxylin and eosin staining revealed adenocarcinoma in specimens obtained from the biliary stricture. (m) This patient received pancreatoduodenectomy, and (n) this patient was diagnosed with distal cholangiocarcinoma.

F‐FB was performed under X‐ray fluoroscopy. The forceps were inserted into the biliary tract, opened at the perihilar side of the stricture, and then drawn into the stricture. The forceps were pressed against the stricture if they became stuck and closed to carry out a biopsy of the tissue. FB‐45Q‐1 (Olympus Optical Co., Ltd.) biopsy forceps, with a 2.6 mm‐diameter cup, were used to carry out a wire‐guided biopsy (Fig. 1). In the F‐FB group, endoscopic sphincterotomy (EST) was carried out for difficult cases, where the endoscopic devices are inserted into the bile duct using a sphincterotome (KD‐V411M‐0725; Olympus Optical Co., Ltd.), if it was not previously performed and was necessary.

POCS was performed using a mini endoscopy (M00546600 SpyGlass DS Access; Boston Scientific Corporation) direct visualization system. A cholangioscope was inserted into the bile duct over the guidewire, and POCS‐FB under direct vision was performed with M00546270 (Boston Scientific Corporation) with a 1.0 mm‐diameter cup (Fig. 1). In the POCS‐FB group, EST was performed for almost all patients who had not previously undergone EST. Patients who received antithrombotic therapy underwent endoscopic papillary balloon dilation (EPBD) using a balloon dilatation catheter (ZR25‐08‐23, RN25‐0630‐18; KANEKA Medix Corporation, Osaka, Japan).

**Figure 2:**
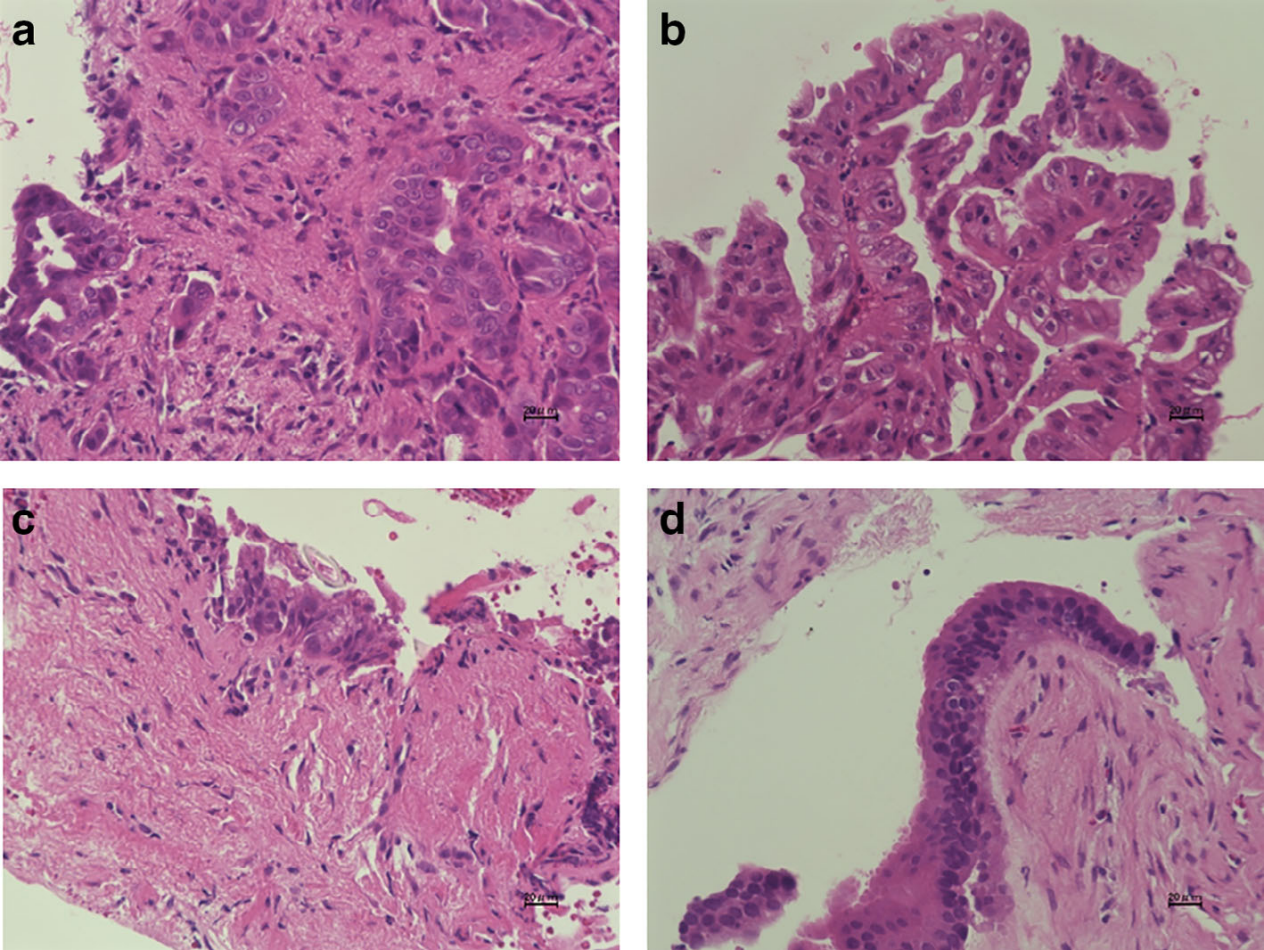
Histopathological findings of specimens obtained by forceps biopsy. (a) A specimen containing biliary epithelial cells with severe cellular and structural atypia is revealed (adenocarcinoma); (b) a specimen with biliary epithelial cells with severe atypia is shown (biliary intraepithelial neoplasia‐3); (c) a specimen containing biliary epithelial cells with mild atypia is revealed (biliary intraepithelial neoplasia‐1); (d) a specimen containing normal biliary epithelial cells is shown.

We also evaluated the adverse events caused by endoscopic procedures and those severities that were referred to as the guidelines of the American Society for Gastrointestinal Endoscopy.

### *Diagnostic criteria*

The diagnosis of cholangiocarcinoma was based on pathological diagnosis of bile aspiration cytology, transpapillary forceps biopsy, endoscopic ultrasonography‐guided fine needle aspiration biopsy, or surgical specimen. Biopsy specimens were stained with hematoxylin and eosin, and if necessary, immunostaining, including Ki‐67 and p53, was also performed. In histological findings, malignancy or suspected malignancy, including biliary intraepithelial neoplasm‐3, was considered positive. Benign and mild atypia was considered negative (Fig. 2). Patients with benign biliary disease had a final diagnosis based on clinical and radiological follow‐up data.

**Figure 3:**
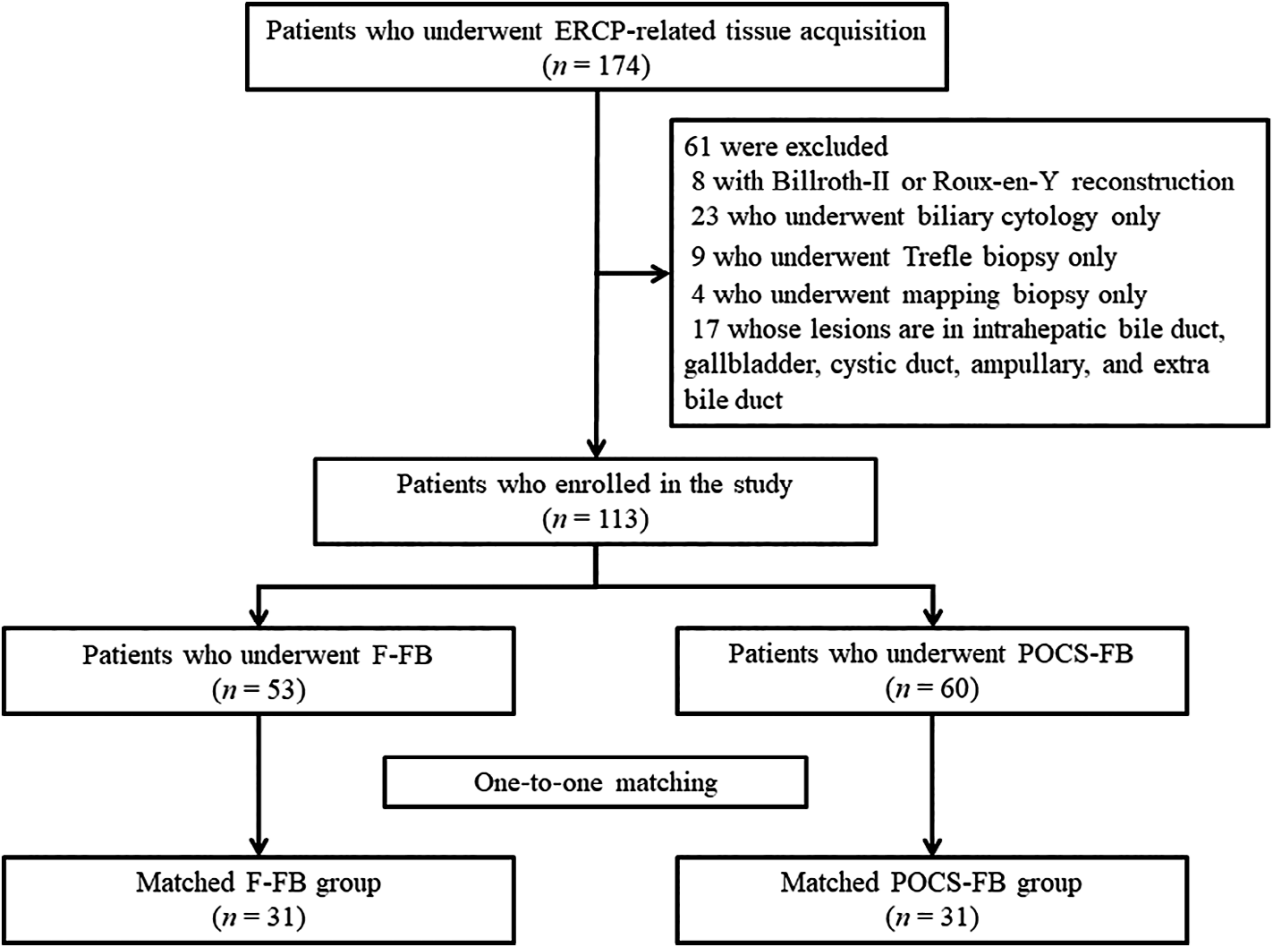
Flowchart of patient division into matched groups of fluoroscopy‐guided forceps biopsy group and peroral cholangioscopy‐guided forceps biopsy group for evaluation of the indeterminate biliary lesions in the study. ERCP, endoscopic retrograde cholangiopancreatography; F‐FB, fluoroscopy‐guided forceps biopsy; POCS‐FB, peroral cholangioscopy‐guided forceps biopsy.

### *Propensity score matching*

We carried out propensity score matching because between‐group differences in baseline characteristics in our study population could influence the diagnostic accuracy of forceps biopsy for ECC. The propensity score of undergoing F‐FB or POCS‐FB was calculated using a multivariable logistic regression model. Length of biliary stricture or tumor size and serum total bilirubin (T‐Bil) were reported as the factors affecting the accuracy or sensitivity of ERCP‐related tissue acquisition for cholangiocarcinoma or malignant biliary stricture.^9^, ^10^, ^11^ Although macroscopic type of cholangiocarcinoma was also reported as a factor affecting the accuracy, it was difficult to accurately differentiate macroscopic type of cholangiocarcinoma before the ERCP procedure, so macroscopic type of cholangiocarcinoma was excluded. In addition, location of biliary lesion, acute cholangitis, level of carcinoembryonic antigen (CEA), level of carbohydrate antigen 19‐9 (CA19‐9), previous EST, and prebiliary stenting before ERCP could determine whether F‐FB or POCS‐FB was necessary. Finally, age and gender were included in the model, along with the following characteristics of patients: age (continuous), gender (male *vs* female), location of biliary lesion (distal *vs* perihilar), length of biliary stricture (continuous), acute cholangitis (present or absent), T‐Bil (continuous), CEA (continuous), CA19‐9 (continuous), previous history of EST (present *vs* absent), and prebiliary stenting before ERCP (done *vs* not done). Each patient in the F‐FB group was matched to a patient in the POCS‐FB group with the nearest‐neighbor method using a caliper range of 0.2 of the standard deviation of the pooled propensity scores.

### *Statistical analysis*

Statistical analysis was performed using StatFlex ver. 7.0 for Windows (Artech Co, Ltd., Osaka, Japan). Categorical variables were compared using the chi‐square test or Fisher's exact test as appropriate. Continuous variables were compared by using the Mann–Whitney *U*‐test. All values are expressed as median with interquartile ranges. *P* < 0.05 was considered significant.

## Results

### *Patient's characteristics and baseline evaluation*

**Figure 4:**
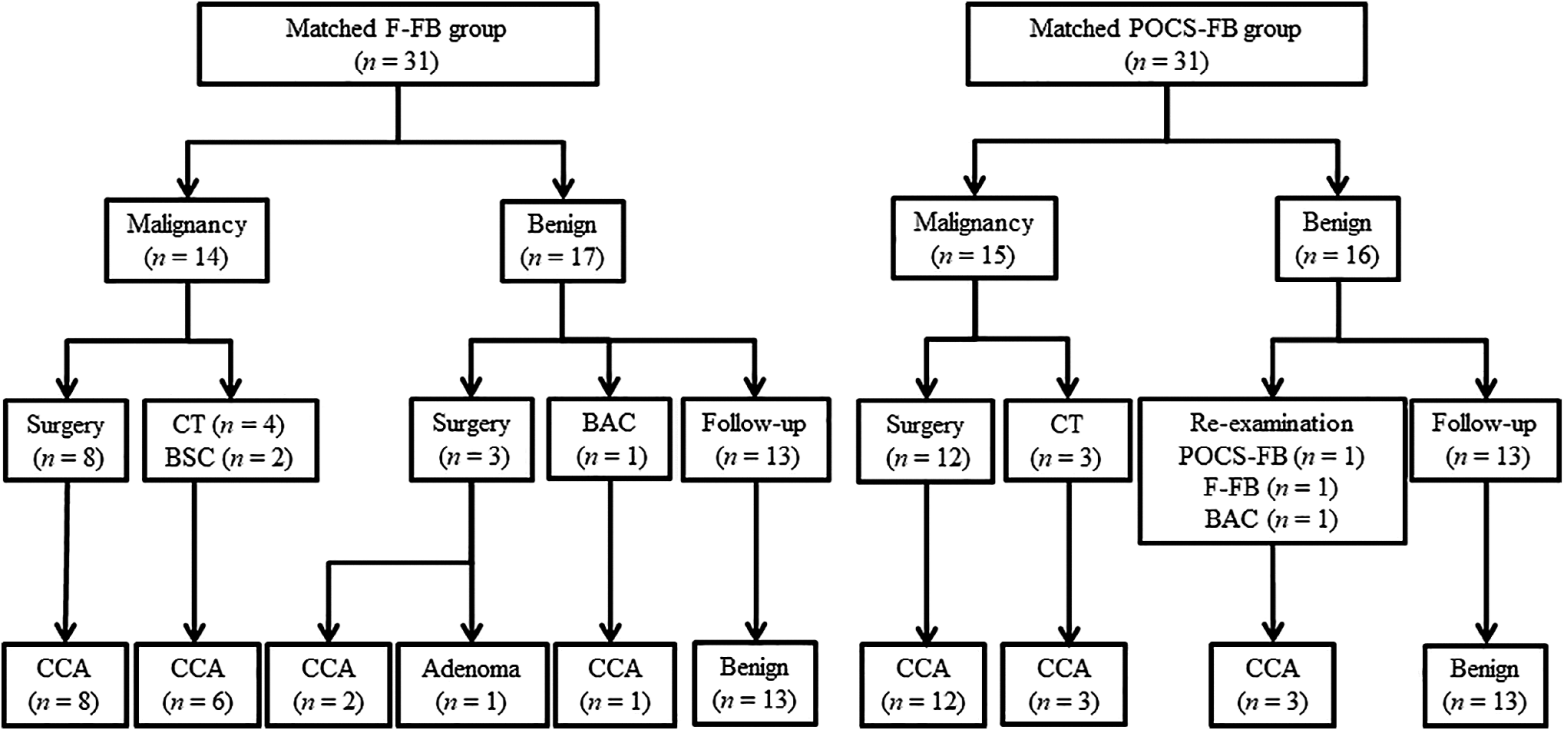
Diagnostic flowchart of the patients in the propensity score‐matched cohort. The final clinical diagnosis was derived from surgical pathology in 23 patients. In both groups, all of the patients diagnosed as malignancy by the histopathological findings of endoscopic tissue acquisitions were cholangiocarcinoma. In the matched fluoroscopy‐guided forceps biopsy (F‐FB) group, 17 patients were diagnosed as benign by the histopathological findings of F‐FB, and 3 of them were finally diagnosed as cholangiocarcinoma by bile aspiration cytology or surgical pathology. In the matched peroral cholangioscopy‐guided forceps biopsy (POCS‐FB) group, 16 patients were diagnosed as benign by the histopathological findings of POCS‐FB, and 3 of them were finally diagnosed as cholangiocarcinoma by re‐examination. BAC, bile aspiration cytology; BSC, best supportive care; CCA, cholangiocarcinoma; CT, chemotherapy; F‐FB, fluoroscopy‐guided forceps biopsy; POCS‐FB, peroral cholangioscopy‐guided forceps biopsy.

We enrolled 113 patients with 114 biliary lesions in this study (Fig. 3). Participants included 75 men and 38 women aged 26–91 years (median age, 73 years). Sixty‐one patients had ECC, and 52 had benign biliary lesions. We performed F‐FB for 53 patients (F‐FB group) and POCS‐FB for 60 patients (POCS‐FB group). Table 1 summarizes the characteristics of all patients in the F‐FB and POCS‐FB groups. Some baseline characteristics, including length of strictures, acute cholangitis, and previous history of EST, were significantly different between the two groups. Propensity score matching balanced these differences between the two groups so that there was no significant difference in age, gender, location of biliary lesions, length of stricture, presence of acute cholangitis, median level of T‐Bil, CEA, and CA19‐9 between the two groups (Table 2). In the propensity score‐matched cohort, the F‐FB group included 10 patients with distal cholangiocarcinoma, 7 patients with perihilar cholangiocarcinoma, and 14 patients with benign biliary strictures. In the F‐FB group, macroscopic types of ECC included 2 papillary type, 11 nodular type, and 4 flat type. The POCS‐FB group included 9 patients with distal cholangiocarcinoma, 9 patients with perihilar cholangiocarcinoma, and 13 patients with benign biliary strictures. In the POCS‐FB group, macroscopic types of ECC included 2 papillary type, 12 nodular type, and 4 flat type. The final clinical diagnosis was derived from surgical pathology in 23 patients (Fig. 4). The median follow‐up periods of benign biliary diseases were 23 months (range, 1–73 months).

**Table 1:** Baseline characteristics of study patients for the fluoroscopy‐guided forceps biopsy group and the peroral cholangioscopy‐guided forceps biopsy group

	F‐FB (*n* = 53)	POCS‐FB (*n* = 60)	*P* value
Age (years)	73 (50–91)	73 (26–88)	0.689
Gender: male/female	31/22	44/16	0.096
Location of biliary lesions (*n* = 114)			
Distal/perihilar/diffuse	31/22/0	29/30/2	0.253
Length of stricture (mm; *n* = 114)	16.3 (3.9–43.3)	14.1 (0–46.0)	0.037
Acute cholangitis (presence/absence)	2/51	10/50	0.026
Total bilirubin (mg/dL)	1.2 (0.5–25.4)	1.2 (0.2–14.8)	0.403
Tumor marker			
CEA (ng/mL)	2.4 (0.8–1549.1)	2.7 (0.8–7616.8)	0.409
CA19‐9 (U/mL)	45.3 (0.8–11 985.0)	24.9 (0.8–9052.0)	0.330
Previous history of EST (presence/absence)	5/48	23/37	<0.001
Prebiliary stenting before ERCP (done/not done)	6/47	15/45	0.062
Disease (*n* = 114)			
Cholangiocarcinoma	29	32	0.612
Intraductal papillary mucinous neoplasm with bile duct	1	1	
Adenoma	1	0	
IgG4‐related sclerosing cholangitis	2	4	
Primary sclerosing cholangitis	2	2	
Drug‐induced cholangitis	0	3	
Peribiliary cyst	0	1	
Right hepatic artery syndrome	1	0	
Benign biliary stricture	17	18	
Macroscopic type (*n* = 61)			
Papillary/nodular/flat	4/19/6	5/21/6	0.989
TNM classification/stage (*n* = 61)			
Distal			
T category is 0/1/2/3/4	1/0/4/11/0	0/2/8/6/1	0.148
N category 0/1/2	11/5/0	11/5/1	0.616
M category 0/1	14/2	15/2	0.948
Stage 0/I/IIA/IIB/IIIA/IIIB/IV	1/0/4/9/0/0/2	0/2/4/8/0/1/2	0.672
Perihilar			
T category is 0/1/2/3/4	0/3/2/5/3	0/2/6/3/4	0.607
N category 0/1/2	7/6/0	11/4/0	0.562
M category 0/1	12/1	12/3	0.353
Stage 0/I/II/IIIA/IIIB/IIIC/IVA/IVB	0/2/1/3/1/5/0/1	0/1/4/1/3/3/0/3	0.597
Procedure time (min)	85 (39–230)	79 (26–170)	0.411
EST (previous or done/not done)	24/29	57/3	<0.001
Number of biopsies, times (*n* = 114)	3 (1–8)	3 (2–8)	0.111
Mapping biopsy (with/without)	16/37	41/19	<0.001

Values are presented as number or median (range).

CA19‐9, carbohydrate antigen 19‐9; CEA, carcinoembryonic antigen; ERCP, endoscopic retrograde cholangiopancreatography; EST, endoscopic sphincterotomy; F‐FB, fluoroscopy‐guided forceps biopsy; POCS‐FB, peroral cholangioscopy‐guided forceps biopsy.

**Table 2:** Baseline characteristics of a propensity score‐matched cohort for the fluoroscopy‐guided forceps biopsy group and the peroral cholangioscopy‐guided forceps biopsy group

	F‐FB (*n* = 31)	POCS‐FB (*n* = 31)	*P* value
Age (years)	71 (50–88)	72 (47–84)	0.983
Gender: male/female	19/12	19/12	1.000
Location of biliary lesions			
Distal/perihilar/diffuse	19/12/0	14/16/1	0.312
Length of stricture (mm)	14.8 (5.4–43.3)	16.8 (0–32.2)	0.961
Acute cholangitis (presence/absence)	1/30	1/30	1.000
Total bilirubin (mg/dL)	1.2 (0.5–25.4)	1.4 (0.2–14.8)	0.905
Tumor marker			
CEA (ng/mL)	2.5 (0.8–10.6)	1.9 (0.8–8.6)	0.314
CA19‐9 (U/mL)	45.3 (0.8–11 985.0)	25.4 (0.8–9052.0)	0.32.1
Previous history of EST (presence/absence)	4/27	2/29	0.671
Prebiliary stenting before ERCP (done/not done)	6/25	5/26	0.740
Disease			
Cholangiocarcinoma	17	18	0.443
IgG4‐related sclerosing cholangitis	0	1	
Primary sclerosing cholangitis	1	1	
Drug‐induced cholangitis	0	2	
Peribiliary cyst	0	1	
Right hepatic artery syndrome	1	0	
Benign biliary stricture	12	8	
Macroscopic type (*n* = 35)			
Papillary/nodular/flat	2/11/4	2/12/4	0.993
TNM classification/stage (*n* = 35)			
Distal			
T category is 0/1/2/3/4	1/0/3/6/0	0/1/4/3/1	0.392
N category 0/1/2	8/2/0	4/4/1	0.228
M category 0/1	10/0	9/0	1.000
Stage I/IIA/IIB/IIIA/IIIB/IV	1/0/3/6/0/0/0	0/1/2/5/0/1/0	0.777
Perihilar			
T category is 0/1/2/3/4	0/0/1/4/2	0/0/5/2/2	0.536
N category 0/1/2	4/3/0	8/1/0	0.347
M category 0/1	7/0	7/2	0.182
Stage I/II/IIIA/IIIB/IIIC/IVA/IVB	0/0/1/2/1/3/0/0	0/0/3/1/2/1/0/2	0.722
Procedure time (min)	88 (39–184)	80 (47–150)	0.735
EST (previous or done/not done)	13/18	29/2	<0.001
Number of biopsies, times	3 (1–8)	3 (2–7)	0.544
Mapping biopsy (with/without)	9/22	23/8	<0.001

Values are presented as number or median (range).

CA19‐9, carbohydrate antigen 19‐9; CEA, carcinoembryonic antigen; ERCP, endoscopic retrograde cholangiopancreatography; EST, endoscopic sphincterotomy; F‐FB, fluoroscopy‐guided forceps biopsy; POCS‐FB, peroral cholangioscopy‐guided forceps biopsy.

The median number of biopsies was three (range, 1–8) and three (range, 2–7) in the F‐FB group and the POCS‐FB group, respectively. In the POCS‐FB group, almost all patients with naïve papilla underwent EST. Meanwhile, only nine patients received EST in the F‐FB group. Therefore, patients who have a previous history of EST or underwent EST in the F‐FB group were significantly fewer than in the POCS‐FB group (*P* < 0.001). The mapping biopsy, a method for defining the longitudinal extension of ECC, was also performed for many patients who underwent POCS‐FB. Meanwhile, fewer patients underwent mapping biopsy in the F‐FB group than that in the POCS‐FB (29.0 *vs* 74.2%, *P* < 0.001). There was no significant difference between the median procedure time of F‐FB and that of POCS‐FB (Table 2).

### *Diagnostic utility of fluoroscopy‐guided forceps biopsy and peroral cholangiography‐guided forceps biopsy for ECC*

Figure 4 shows a diagnostic flowchart of the propensity score‐matched cohort. The diagnostic yields of F‐FB and POCS‐FB to differentiate cholangiocarcinoma from benign biliary disease are shown in Table 3. The values for sensitivity, specificity, positive predictive value (PPV), negative predictive value (NPV), and accuracy of F‐FB were 82.4, 100, 100, 82.4, and 90.3%, respectively, and for POCS‐FB, the values for sensitivity, specificity, PPV, NPV, and accuracy of POCS‐FB were 83.3, 100, 100, 81.3, and 90.3%, respectively. There was no significant difference in the sensitivity, specificity, PPV, NPV, and accuracy of F‐FB and POCS‐FB for ECC in the propensity score‐matched cohort.

**Table 3:** Diagnostic performance of the propensity score‐matched cohort for the fluoroscopy‐guided forceps biopsy group and the peroral cholangioscopy‐guided forceps biopsy group

	F‐FB (*n* = 31)	POCS‐FB (*n* = 31)	*P* value†
Sensitivity (%)	82.4 (14/17)	83.3 (15/18)	1.000
Specificity (%)	100 (14/14)	100 (13/13)	1.000
Positive predictive value (%)	100 (14/14)	100 (15/15)	1.000
Negative predictive value (%)	82.4 (14/17)	81.3 (13/16)	1.000
Accuracy (%)	90.3 (28/31)	90.3 (28/31)	1.000

† *P* value; Fisher's exact test.

F‐FB, fluoroscopy‐guided forceps biopsy; POCS‐FB, peroral cholangioscopy‐guided forceps biopsy.

### *Adverse events*

Adverse events following F‐FB occurred in 13 patients (41.9%), with 9 patients developing acute pancreatitis (29.0%), 3 patients developing infection (cholangitis, 9.7%), and 1 developing pulmonary disorder (3.2%). Adverse events following POCS‐FB occurred in nine patients (29.0%), with six patients developing acute pancreatitis (19.4%), including one of severe pancreatitis; two developing infections (cholangitis, 6.5%); and one patient who underwent a precut papillotomy developing bleeding (3.2%). There was no significant difference in the occurrence of adverse events between the F‐FB group and the POCS‐FB group. No perforations and no procedure‐related mortality were observed.

## Discussion

To obtain the specimens from the biliary lesions, ERCP‐related tissue acquisition, including bile aspiration cytology, biliary brush cytology, and forceps biopsy under X‐ray fluoroscopy, is a commonly used method. Every tissue acquisition method is almost 100% specific.^5^ However, the sensitivities of bile aspiration cytology, biliary brush cytology, and forceps biopsy for malignant biliary lesions, reported as 41.6, 45.0, and 48.1%, respectively, were insufficient.^4^, ^5^ A combination of biliary brush cytology and forceps biopsy only modestly increased the sensitivity to 59.4%.^5^ Although ERCP plays important roles in the biliary drainage for biliary lesions with obstructive jaundice, in diagnosing indeterminate biliary lesions, it is inadequate for clinical use because of the poor sensitivity of ERCP‐related tissue acquisition for malignant biliary strictures.

Recently, POCS has been widely used in diagnosing indeterminate biliary lesions. We can not only observe the optical viewing of the biliary systems but also perform targeted biopsies on indeterminate biliary lesions under direct vision with POCS. In previous studies, the use of POCS improved the diagnostic accuracy of indeterminate biliary lesions.^12^, ^13^ Although the high sensitivity of visual diagnosis by POCS for malignant biliary strictures was reported (86.7%), its specificity was insufficient (71.2%).^14^ Therefore, histological diagnosis, including POCS‐FB, for indeterminate biliary lesions is needed. However, the sensitivities of POCS‐FB for malignant biliary strictures and for cholangiocarcinoma were also insufficient (60.1 and 66.2%, respectively).^6^ On the other hands, some studies reported that F‐FB and POCS‐FB are equally limited in establishing the diagnosis of malignancy in indeterminate biliary lesions.^7^, ^8^ It is still unknown which technique was superior to the other. Therefore, we compared the diagnostic ability of F‐FB and that of POCS‐FB. In our study, there was no significant difference between the diagnostic accuracy of F‐FB and that of POCS‐FB. Although POCS allows targeted biopsies under optical viewing, the specimen obtained by POCS is relatively small. Hartman *et al*. reported that less tissue was obtained from POCS‐FB than F‐FB.^8^ It was a possible reason for the lack of sensitivity in the pathological diagnostic ability of POCS‐FB. In clinical practice, some specimens obtained by POCS‐FB were so small that the results of the pathological examination were indeterminate or had insufficient material. We also experienced a patient who could not be diagnosed accurately by POCS‐FB. This patient received re‐ERCP with F‐FB and had a final diagnosis of perihilar cholangiocarcinoma. Although we performed multiple biopsies (median number of biopsies, 3; range, 2–7) for all cases with indeterminate biliary lesions, the sensitivities of POCS‐FB for cholangiocarcinoma were also insufficient (83.3%) in the study. Therefore, biopsies repeated four times or more might be needed to increase the diagnostic accuracy of POCS‐FB for cholangiocarcinoma. Some combinations of other tissue acquisition methods, such as F‐FB, should be performed as much as possible if the cost and the additional endoscopic procedure time are acceptable. In the future, to improve the diagnostic ability of POCS‐FB for indeterminate biliary lesions, a new peroral cholangioscope system with a larger‐diameter working channel for large‐capacity forceps might be needed.^15^

In our study, almost all patients (93.5%) had a previous history of EST or underwent EST in POCS‐FB, and this rate was significantly higher than that in F‐FB. For biliary access, EST are is when the cholangioscope is inserted into the bile duct, but as a result, the sphincter of Oddi function is lost entirely. Although whether EST is or not needed to insert a biopsy forceps into the bile duct for the F‐FB is controversial, avoiding EST may help prevent early adverse events, including bleeding, and late adverse events, including liver abscess.^16^, ^17^

Longitudinal extension is a feature of cholangiocarcinoma, and this was used in 14.6% of patients with cholangiocarcinoma.^18^, ^19^ To avoid positive resection margins after surgery, preoperative identification of the exact perihilar and distal margins of resectable ECC is essential. In both fluoroscopy and POCS, the mapping biopsy was also reported as a useful technique for the preoperative assessment of the longitudinal extension of ECC.^20^, ^21^, ^22^ Although it is uncertain whether POCS‐guided forceps mapping biopsy was superior or not to fluoroscopy‐guided mapping biopsy for defining the longitudinal extension of ECC, we selected the POCS technique rather than the fluoroscopy‐guided technique to perform the mapping biopsy. Visual findings of POCS also had high accuracy (76.9–83.7%) when evaluating the longitudinal extension of ECC,^13^, ^14^, ^23^ and it might why we selected the POCS technique for the mapping biopsy. During the study period, 19 patients with 20 lesions underwent surgical resection for ECC (distal cholangiocarcinoma, 16; perihilar cholangiocarcinoma, 4) after POCS‐guided forceps mapping biopsy, 14 patients underwent pancreatoduodenectomy, 3 underwent hepatectomy with extrahepatic bile duct resection, 1 received pancreatoduodenectomy with hepatectomy, and 1 received extrahepatic bile duct resection with cholecystectomy in our study. Five patients had surgical margins that were positive at the hepatic side, and for all of them, the diagnostic accuracy of POCS‐guided forceps mapping biopsy for the lateral extension of ECC could not be evaluated. Fourteen patients did not undergo POCS‐guided forceps mapping biopsy to evaluate the lateral extension at the duodenal side. The specimens obtained from the hepatic side of ECC were insufficient in a patient, and those from the duodenal side were also insufficient in two patients. The diagnostic accuracy of the lateral extent at the hepatic side and the duodenal side were 85.7% (12/14) and 40.0% (2/5), respectively. However, considering the extensive cost of POCS, at least the initial diagnostic technique based on pathological evaluation for indeterminate biliary lesions might have to be performed using F‐FB, which has similar accuracy to POCS‐FB. The indications of POCS‐FB might be limited for the cases that could not be diagnosed by F‐FB and/or the cases that required POCS‐guided forceps mapping biopsy to evaluate the exact range of resectable ECC.

This study has some limitations. First, this was a single‐center study with a small number of cases. Second, patients who were diagnosed by a clinical follow‐up were also included in this study. A prospective randomized study including a larger number of patients with long‐term follow‐up is required.

In conclusion, the diagnostic performance of F‐FB for ECC is similar to that of POCS‐FB. At least for the initial diagnostic technique based on pathological evaluation for indeterminate biliary lesions, POCS‐FB is not necessary.
